# *Cedecea davisae'*s Role in a Polymicrobial Lung Infection in a Cystic Fibrosis Patient

**DOI:** 10.1155/2012/176864

**Published:** 2012-12-23

**Authors:** Thayer G. Ismaael, Eleana M. Zamora, Faisal A. Khasawneh

**Affiliations:** ^1^Department of Internal Medicine, Texas Tech University Health Sciences Center, Amarillo, TX 79106, USA; ^2^Division of Pulmonary, Critical Care and Sleep Medicine, Department of Internal Medicine, University of New Mexico, Albuquerque, NM 87131, USA; ^3^Section of Infectious Diseases, Department of Internal Medicine, Texas Tech University Health Sciences Center, Amarillo, TX 79106, USA

## Abstract

Chronic airway colonization and infection are the hallmark of cystic fibrosis (CF). *Staphylococcus aureus, Pseudomonas aeruginosa*, and *Burkholderia cepacia* are well-documented bacterial culprits in this chronic suppurative airway disease. Advanced molecular diagnostics have uncovered a possible role of a larger group of microorganisms in CF. *Cedecea* is a member of the family Enterobacteriaceae and is an emerging pathogen. We present a case of a polymicrobial healthcare-associated pneumonia in a CF patient caused by *Cedecea davisae*, among other bacteria.

## 1. Background

Cystic fibrosis (CF) is an autosomal recessive disorder characterized by viscous secretions [1]. Tenacious secretions render mucus-clearing mechanisms and cough ineffective and predispose to the development of bronchiectasis. Furthermore, CF is characterized by episodic exacerbations and acute pulmonary infections. 

Most respiratory bacterial infections in CF patients are caused by *Staphylococcus aureus*, *Pseudomonas aeruginosa*, and *Burkholderia cepacia* complex [2]. Recent advances in molecular diagnostics have raised the possibility of more diverse bacterial infections, including anaerobes and Enterobacteriaceae [3]. Although these bacteria might play a role in exacerbations and acute pneumonias in this group of patients, they are not as persistent in the CF lung as the aforementioned bacteria. 

*Cedecea davisae* is an emerging pathogen [4, 5]. It is a Gram-negative bacterium that has been implicated in causing catheter-related blood stream infection, bacteremic skin and soft tissue infection, and lung infection [4–6]. 

We present a case of a polymicrobial pulmonary infection in a CF patient caused by methicillin-susceptible *S. aureus *(MSSA), *B. cepacia,* and *C. davisae*. The latter has not been reported in CF patients, nor has it been demonstrated to be as extensively drug resistant as in the case at hand. 

## 2. Case Presentation 

A 20-year-old female patient with CF presented with 5-day history of fevers, progressive shortness of breath, and productive cough with increasing sputum amount and purulence. In the past 2 years, the patient had an average of 5 or 6 admissions per year for CF exacerbations. She was known to be colonized by multidrug-resistant (MDR) *P. aeruginosa* and Methicillin-resistant *S. aureus* (MRSA). She had an extensive exposure to all classes of antibiotics, including carbapenems and intravenous as well as nebulized colistin. The patient reported a sulfa allergy since childhood. 

**Figure 1:**
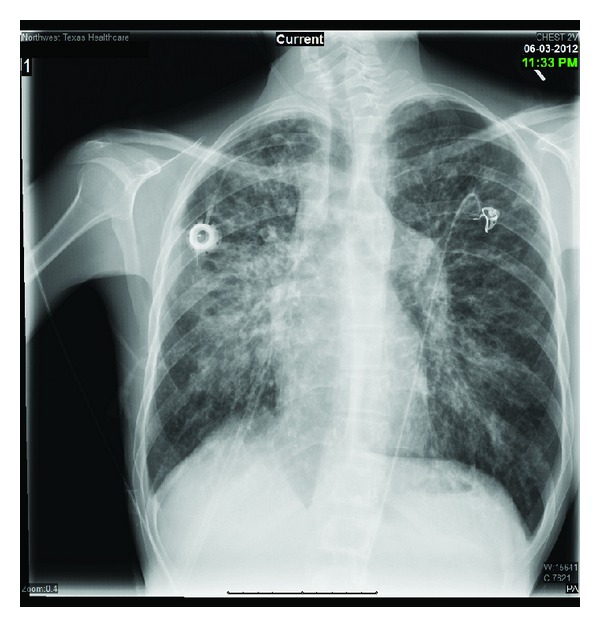
Admission chest X-ray showing right perihilar alveolar infiltrates superimposed on chronic pulmonary infiltrates.

On examination, the patient had severe cachexia and was in obvious respiratory distress. Temperature was 98.4 Fahrenheit (F°) and her lungs exam revealed bilateral coarse crackles. Her white blood cell count was 19.0 × 10^3^ with 32% bands and her admission chest X-ray showed right perihilar alveolar infiltrates superimposed on chronic CF changes (Figure 1).

**Figure 2:**
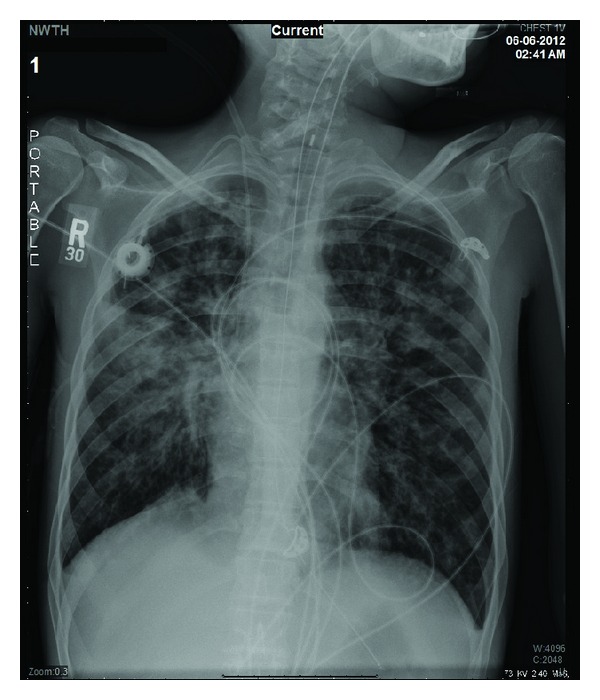
Chest X-ray on ICU admission.

After collecting appropriate samples, the patient was started on linezolid, meropenem, and nebulized colistin and was admitted to the intermediate care unit with a suspected healthcare-associated pneumonia (HCAP). The patient's condition continued to worsen, requiring intensive care unit (ICU) transfer on her third hospital day. At that time, her temperature was 100.3 F° and she was in severe respiratory distress. The chest X-ray showed persistent right perihilar infiltrates (Figure 2). She underwent intubation and mechanical ventilation (MV) in addition to vasopressor support. The blood cultures grew MSSA and the sputum culture showed a heavy growth of the following bacteria: MSSA, *B. cepacia, and C. davisae*. Bacterial identification and susceptibility testing were performed using MicroScan WalkAway 40 plus (Siemens Healthcare Diagnostics Inc., West Sacramento, CA). The *B. cepacia* was resistant to all tested antibiotics except for trimethoprim/sulfamethoxazole (TMP/SMX). The *C. davisae* was resistant to all tested antibiotics, including all beta-lactams, aminoglycosides, fluoroquinolones, and tigecycline, but it was susceptible to TMP/SMX. Susceptibility testing was performed based on the Clinical and Laboratory Standards Institute (CLSI) guidelines. 

Given the patient's reported sulfa allergy, a rapid TMP/SMX desensitization protocol was initiated. She was able to tolerate full dose of TMP/SMX (20 milligrams/kilograms of TMP/day). During this time, no other changes in her antibiotic regimen were undertaken. Subsequently, the patient's condition started to improve. Follow up blood cultures were negative. She came off vasopressor support and was successfully liberated from MV on her eighth hospital day. Three days later, she was transferred to a regular floor where she finished a 4-week course of meropenem, TMP/SMX, and nebulized colistin. The patient was discharged home off antibiotics and on no supplemental oxygen therapy. 

## 3. Discussion

The HCAP in the presented case is polymicrobial. The role of MSSA was indisputable given that the bacteria grew in sputum and blood cultures. The fact that the patient's condition worsened despite adequate treatment for MSSA and the improvement that followed the addition of TMP/SMX proved that *B. cepacia* and *C. davisae* played a role in this infection. The literature supports *B. cepacia*'s role as a colonizer and potential pathogen in CF but the cases reviewed below make it hard to ignore *C. davisae*'s role in this medically compromised CF patient [7–10]. 

The genus *Cedecea* represents Gram-negative, oxidase-negative, fermentative bacilli that belong to the family Enterobacteriaceae [9]. Strains of Cedecea resemble those of *Serratia*; both are lipase positive and intrinsically resistant to colistin and cephalothin. Cedecea was formerly known as CDC enteric group 15 and was designated as a new and separate genus in 1981. The name was constructed from the abbreviation CDC (Centers for Disease Control). Cedecea has 5 species (*spp*.); *C. davisae*, *C. lapagei*, *C. netri*, Cedecea *spp. *3, and Cedecea *spp.* 5 [7]. 

Cedecea species have environmental reservoirs including aqueous bodies [6]. More than 50% of the isolates from agricultural dust were of the Enterobacteriaceae family and of this 0.7% were Cedecea [11]. The highest numbers were found in association with maize and sorghum and it is possible that house dust may harbor this organism. Cedecea is well known to colonize the gut and potentially the airways but it is not a known member of the human skin flora [10]. It has been isolated from blood, sputum, skin, and mucus membrane ulcers and gastrointestinal tract samples. However, infections due to these bacteria are rarely reported [12–16]. 

A literature search revealed 8 reported cases of *C. davisae* infections (Table 1). Upon reviewing those cases the following was noticeable.

- All affected patients were either elderly or immunocompromised or had multiple comorbidities which support the possibility of Cedecea being an opportunistic pathogen.
- Some infections were community acquired, like the 67-year-old diabetic who was admitted with a bacteremic leg infection; meanwhile others were nosocomial, like the 52-year-old male with acute myeloid leukemia who developed catheter-related blood stream infection on his 25th day of hospitalization.
- The outcomes were favorable and the infection resolved in all reported cases.
- None of the reported *C. davisae* was as extensively resistant as the case at hand, which demonstrates the ability of these bacteria to adapt and acquire different resistance mechanisms.

Our case underscores the increasing recognition of emerging and potentially opportunistic microorganisms like Cedecea species in the constantly growing medically compromised patients. Further work is needed to identify these bacteria's ecological niche, mode of transmission, infectious spectrum, and best treatment options. 

## Figures and Tables

**Table 1:** Reported cases of *C. davisae* infections.

Age (yrs)/gender	Comorbidities	Infection	Cultured samples	Sensitivity among tested drugs	Reference number
20/F	CF, DM	HCAP	Sputum	TMP/SMX only	Current case
54/M	Colon cancer	Bacteremia	Blood	cefoxitin, ciprofloxacin, ceftriaxone, ceftazidime, imipenem, gentamicin, aztreonam	[7]
52/M	AML	CRBSI	Blood	Aminoglycosides, cefepime, imipenem	[4]
42/M	DM, Kidney transplantation	Infected oral ulcer	Oral ulcer	Fluoroquinolones, TMP/SMX, cefepime, ceftazidime, ceftriaxone, gentamicin, cefotetan, ampicillin	[8]
67/M	DM, leg ulcer	DFI	Blood, leg ulcer	Aminoglycosides, meropenem, ceftazidime, cefotaxime, cefepime, aztreonam, amoxicillin/clavulanic acid, TMP/SMX, ciprofloxacin	[6]
70/F	COPD, CHF	CRBSI	Blood	Aminoglycoside, carbenicillin, cefotaxime, cefoperazone, piperacillin	[9]
50/M	CHF, alcoholic hepatitis	SSTI	Scrotal abscess	Ampicillin, cephalothin, carbenicillin, tetracycline, aminoglycosides	[10]
76/M	CHF, DM	Pneumonia	Sputum	Aminoglycosides, carbenicillin, cefamandole, chloramphenicol, tetracycline, TMP/SMX	[5]
65/F	HTN, atherosclerosis	Pneumonia	Sputum	No data	[5]

Abbreviations: AML: acute myeloid leukemia; CF: cystic fibrosis; CHF: congestive heart failure; COPD: chronic obstructive pulmonary disease; CRBSI: catheter-related blood stream infection; DFI: diabetic foot infection; DM: diabetes mellitus; F: female; HCAP: healthcare-associated pneumonia; HTN: hypertension; M: male; SSTI: skin and skin structure infection; TMP/SMX: trimethoprim/sulfamethoxazole; yrs: years.
